# New DSM-5 maladaptive symptoms in PTSD: gender differences and correlations with mood spectrum symptoms in a sample of high school students following survival of an earthquake

**DOI:** 10.1186/s12991-014-0028-9

**Published:** 2014-11-18

**Authors:** Claudia Carmassi, Paolo Stratta, Gabriele Massimetti, Carlo Antonio Bertelloni, Ciro Conversano, Ivan Mirko Cremone, Mario Miccoli, Angelo Baggiani, Alessandro Rossi, Liliana Dell’Osso

**Affiliations:** Section of Psychiatry, Department of Clinical and Experimental Medicine, University of Pisa, Pisa, Italy; Section of Psychiatry, Department of Experimental Medicine, University of L’Aquila, L’Aquila, Italy; Unit of Hospital Hygiene and Epidemiology, Department of Traslational Research, University of Pisa, Pisa, Italy

**Keywords:** Trauma, Risk-taking behaviors, maladaptive behaviors, Post-Traumatic Stress Disorder (PTSD), Bipolar Disorder

## Abstract

**Background:**

Gender differences in post-traumatic stress disorder (PTSD) rates were confirmed across different DSM editions as well as the role of bipolar disorder (BD) comorbidity on prevalence and course, but little data is available upon new DSM-5 criteria, including maladaptive behaviors. The aim of this study was to investigate gender differences in DSM-5 PTSD in a sample of young adult earthquake survivors and the impact of lifetime mood spectrum comorbidity.

**Methods:**

Five hundred twelve young adult survivors from the L’Aquila 2009 earthquake were evaluated by Trauma and Loss Spectrum-Self Report (TALS-SR) and Mood Spectrum-Self Report (MOODS-SR).

**Results:**

Females showed significantly higher DSM-5 PTSD prevalence rates than men. Similarly, female survivors with DSM-5 PTSD showed significantly higher scores in several of the MOODS-SR and TALS-SR domains with respect to males. Males showed significantly higher scores in the TALS-SR *maladaptive coping* domain only. A significant positive association between the MOODS-SR manic-hypomanic component and TALS-SR *potentially traumatic events* and *maladaptive coping* domains emerged in the whole sample, particularly among men.

**Conclusion:**

This study allows a first glimpse on gender differences in DSM-5 PTSD criteria in a sample of earthquake survivors. Further, possible correlations with subthreshold manic-hypomanic comorbidity are suggested among males, showing a significant trend particularly for lifetime trauma exposure and for the newly introduced maladaptive behaviors.

## Background

Post-Traumatic Stress Disorder (PTSD) is a highly debilitating disorder that represents one of the most frequent consequences of the exposure to mass trauma, particularly earthquakes [1-10].

Since its first appearance in the DSM-III [11], PTSD diagnostic criteria have been changed across the subsequent editions of the Diagnostic and Statistical Manual (DSM), accordingly to increasing clinical and research findings in different populations. The last edition of the DSM (DSM-5) [12], just published in May 2013, introduced some important changes for what concern PTSD diagnosis. Besides defining a four-symptom cluster structure, instead of the DSM-IV-TR three one [13], the DSM-5 introduced three new symptoms: “persistent, distorted cognitions about the cause or consequences of the traumatic event(s) that lead the individual to blame himself/herself or others” (D3) and “persistent negative emotional state (e.g., fear, horror, anger, guilt, or shame)” (D4), added in criterion D, exploring negative alterations in cognition and mood; “reckless or self-destructive behavior” (E2), added in criterion E, addressing symptoms of hyper-arousal. The introduction of this latest symptom, in particular, is related to the increasing evidence emerged in the last decades on the role of maladaptive behaviors in PTSD.

Maladaptive behaviors have been defined as volitional behaviors whose outcome is uncertain and which entail negative consequences that impact everyday activities [14-18]. The role of these behaviors in PTSD patients, like risk-taking behaviors, dangerous driving, or promiscuous sex, is evidenced by an increasing amount of literature [7,19-23]. Significantly higher rates of aggressive traits, including violent behaviors were found in Iraq and Afghanistan war veterans with PTSD, compared to those without PTSD [24,25]. Male veterans also showed aggressive and unsafe driving [26,27]. Even the medication non-adherence rate resulted higher in veterans with PTSD with respect to those without [28]. Adolescents and young adults with PTSD due to terrorism, fire, or violence, showed risk-taking behaviors, such as smoking, alcohol and substance use, car racing, weapon carrying, violence, and delinquency. Some studies reported higher rates of maladaptive behaviors in victims with PTSD compared to non-affected subjects, with boys reporting significantly higher rates than girls [29-32].

In a previous study on young adult survivors of the 2009 earthquake that stroke the town of L’Aquila (Richter magnitude 6.3), causing 309 deaths, with about 1.600 injured individuals and 66.000 displaced, some of us reported significantly higher maladaptive coping prevalence rates among subjects with DSM-IV-TR PTSD, particularly males [18]. In a recent study [10], we further confirmed relevant rates of endorsement of the new E2 symptom among PTSD L’Aquila survivors diagnosed accordingly to DSM-5 criteria. In both studies, maladaptive behaviors had been explored by means of a spectrum approach to PTSD that explores a broad array of symptoms related to the Axis I disorder, including behaviors and personal characteristics that might represent manifestations and/or risk factors for the development of a stress response syndrome that also address these recently introduced criterion symptoms [9,10].

Several authors have recently indicated the relationship between PTSD and bipolar disorders (BD). BD patients demonstrated to be at higher risk for trauma exposure [33-37] and, when exposed, to be more vulnerable to developing PTSD. Rates of trauma exposure as high as 98% were reported in BD patients, compared to 51%–61% of the general population, suggesting a role of this disorder in increasing the risk of trauma exposure [34]. The National Comorbidity Survey (NCS) reported a lifetime diagnosis of PTSD in 38.8% of individuals with bipolar I disorder [38,39]. Further studies showed that the prevalence of PTSD is increased in bipolar patients [35,40-45]. In previous studies, exploring Italian PTSD patients, a correlation was found between lifetime subthreshold manic-hypomanic and depressive symptoms and both the likelihood of suicidal ideations or attempts [46].

The aims of this study were to explore, in a sample of high school students survived from the L’Aquila earthquake 21 months earlier, the relationships between manic-hypomanic spectrum symptoms and post-traumatic spectrum symptoms and the gender differences in these relationships.

## Methods

### Study participants

The study sample included 512 high school seniors (280 males and 232 females) from L’Aquila (Italy). All subjects were living in the town of L’Aquila at the time the earthquake occurred; all of them were exposed to the April 6, 2009 earthquake. Assessments were performed 21 months after the exposure.

Out of the 10 high school students in L’Aquila, 3 were chosen with technical, scientific, and humanistic orientation programs, respectively. No a priori inclusion or exclusion criteria were used. These schools have students with wide and representative socioeconomic backgrounds. The study was approved by the Ethical Committee of the University of L’Aquila and the school council approved all recruitment and assessment procedures. The study was carried out in accordance with the Declaration of Helsinki. Eligible subjects provided written informed consent, after receiving a complete description of the study and having the opportunity to ask questions. Subjects were not paid for their participation in accordance with the Italian law for clinical studies.

### Instruments and assessments

Assessment instruments included the Trauma and Loss Spectrum-Self Report (TALS-SR) [47,48] and the Mood Spectrum-Self Report (MOODS-SR) [49], lifetime versions. These instruments were developed by the authors who comprise the Italian–American team of researchers belonging to the so called *Spectrum Project*: an international collaboration research project between researchers of the Universities of Pisa (Italy), Pittsburgh (USA), Columbia (New York, USA), and California at San Diego (USA), established to develop and test assessment instruments for the assessment of the spectrum of clinical features associated with the current version of DSM psychiatric disorders. The spectrum model highlights the significance of isolated symptoms and subthreshold symptom clusters that accompany each disorder classified in the Diagnostic and Statistical Manual of Mental Disorders (DSM) and may follow or be manifested in concurrence with the main disorder [50,51]. Originally developed in English, the interviews and self-reports were then translated into Italian, back translated, and then revised for inconsistencies between the two languages. Spectrum assessments for the major mental disorders have been developed [48,49,52-56]. In the present study, we used the final Italian version of the TALS-SR and MOODS-SR.

The TALS-SR is a questionnaire developed for assessing post-traumatic spectrum symptoms [48]. It includes 116 items exploring the lifetime experience of a range of loss and/or traumatic events and lifetime symptoms, behaviors, and personal characteristics that might represent manifestations and/or risk factors for the development of a stress response syndrome. The instrument is organized into nine domains including: *loss events* (I); *grief reactions* (II); *potentially traumatic events* (III); *reactions to losses or upsetting events* (IV); *re-experiencing* (V); *avoidance and numbing* (VI); *maladaptive coping* (VII); *arousal* (VIII); and *personal characteristics/risk factors* (IX). Domain VII, *maladaptive coping*, specifically investigates maladaptive coping and behaviors including no self-care, scarce adherence to ongoing medications, alcohol or drug abuse, risk-taking behaviors (dangerous driving, promiscuous sex, etc.), thoughts of death, and suicidal ideations and attempts. The responses to the items are coded in a dichotomous way (yes/no) and domain scores are obtained by counting the number of positive answers. In line with previous studies, symptom criteria were derived from the positive answers to the TALS-SR items corresponding to the DSM-5 criteria for PTSD [7,8,10].

The MOODS-SR consists of 161 items coded as present or absent for one or more periods of at least 3–5 days throughout the subject’s lifetime. The items are organized into manic and depressive components as well as into a section that assesses disturbances in rhythmicity and vegetative functions, yielding a total of seven domains. In fact, both the manic and the depressive components are subtyped into three domains exploring mood, energy, and cognition symptoms, respectively. The number of the mood-, energy-, and cognition-manic items endorsed by subjects makes up the *manic component* (62 items) while the sum of the mood-, energy-, and cognition-depressive items constitutes the *depressive component* (63 items). The rhythmicity and vegetative function domain (29 items) explores alterations in the circadian rhythms and vegetative functions, including changes in energy, physical well-being, mental and physical efficiency related to the weather and season, and changes in appetite, sleep, and sexual activities.

### Statistical analyses

Mann–Whitney test was computed in order to compare MOODS-SR and TALS-SR domain scores as they are not normally distributed.

Multiple linear regression models were utilized to study the relationships between MOODS-SR depressive and manic-hypomanic components and TALS-SR maladaptive symptoms.

Scattergrams, univariate linear regressions, and coefficients of determination were utilized to evaluate the specific relationship between the MOODS-SR manic-hypomanic component and the TALS-SR maladaptive coping domain in PTSD by gender and non-PTSD by gender subgroups. Further, *t*-tests were computed to compare the slopes of the regression lines.

Eight point biserial correlation coefficients were computed to study the strength of association between MOODS-SR manic-hypomanic component and each TALS-SR maladaptive coping domain item in PTSD by gender subgroups.

Finally, *χ*^2^ tests were computed in order to compare the rates of endorsement of the TALS-SR maladaptive coping items between the two genders.

## Results

Full data were available for 475 young adults (94.2% of the overall sample, mean age 17.67 ± 0.78), 203 women and 272 men. Among the 475 young adults enrolled, 169 (35.6%) subjects presented a diagnosis of PTSD according to DSM-5 [13], with significantly higher rates in females than in males (*n* =104; 51.2% vs *n* =65; 23.9%, respectively, *p* < .001).

All MOODS-SR and TALS-SR domain scores were significantly higher in survivors with DSM-5 PTSD with respect to those without (*p* < .001). Among survivors with DSM-5 PTSD, females reported significantly higher scores in MOOD-SR *mood-depressive* (*p* = .045) and *rhythmicity and vegetative function* (*p* = .027) domains and in TALS-SR *loss events* (*p* = .004), *reactions to losses or upsetting events* (*p* = .001), *re-experiencing* (*p* = .002), and *arousal* (*p* < .001) domains with respect to males. Conversely, the males showed significantly higher scores than females in the TALS-SR *maladaptive coping* domain only (*p* = .012) (Table 1).

**Table 1:** **MOODS-SR and TALS-SR domain scores in 475 L’Aquila survivors with DSM-5 PTSD: gender differences**

	**Total**	**Males**	**Females**	***p***
	**mean ± SD**	**mean ± SD**	**mean ± SD**	
**MOODS-SR**				
Mood-depressive	11.36 ± 5.13	10.25 ± 5.43	12.06 ± 4.82	.045
Mood-manic	12.73 ± 4.96	13.22 ± 5.62	12.43 ± 4.49	.558
Energy-depressive	3.93 ± 2.20	3.55 ± 2.26	4.17 ± 2.14	.090
Energy-manic	5.40 ± 2.58	5.11 ± 2.80	5.59 ± 2.42	.320
Cognition-depressive	10.02 ± 5.48	9.72 ± 6.29	10.20 ± 4.93	.434
Cognition-manic	7.20 ± 4.59	7.78 ± 5.40	6.84 ± 3.99	.414
Rhythmicity	12.02 ± 5.42	10.71 ± 6.27	12.85 ± 4.66	.027
Total depressive	25.31 ± 11.10	23.52 ± 11.61	26.43 ± 10.67	.059
Total manic	25.34 ± 9.96	26.11 ± 11.20	24.85 ± 9.13	.643
**TALS-SR**				
Loss events	4.33 ± 1.72	3.85 ± 1.72	4.63 ± 1.67	.004
Grief reactions	12.66 ± 5.37	11.92 ± 5.50	13.12 ± 5.27	.166
Potential traumatic events	4.73 ± 2.54	4.80 ± 2.88	4.68 ± 2.31	.923
Reactions to losses	10.14 ± 3.03	9.12 ± 2.86	10.78 ± 2.96	.001
Re-experiencing	5.08 ± 1.71	4.59 ± 1.78	5.41 ± 1.59	.002
Avoidance and numbing	5.88 ± 1.69	5.64 ± 1.71	6.03 ± 1.67	.096
Maladaptive coping	1.52 ± 1.47	1.85 ± 1.49	1.32 ± 1.42	.012
Arousal	3.34 ± 1.20	2.75 ± 1.22	3.71 ± 1.03	<.001

Eight multiple linear regression models were applied to the PTSD subgroup to explore the possible relationship between the MOODS-SR depressive and manic-hypomanic components (assumed as predictors) and each of the eight TALS-SR domains (assumed as dependent variables). A significant positive association emerged between the MOODS-SR depressive component and each one of the TALS-SR domains; conversely the MOODS-SR manic-hypomanic component had a significant positive association with TALS-SR *potentially traumatic events* [*b* =0.06, (SE =0.01), *p* < .001] and *maladaptive coping* [*b* =0.02, (SE =0.01), *p* = .031] domains only.

**Figure 1:**
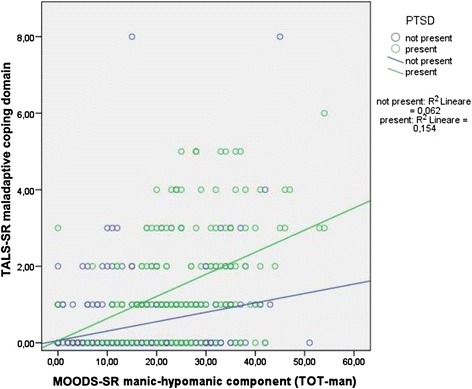
**Relationships between MOODS-SR manic-hypomanic component and TALS-SR maladaptive coping domain in subjects with and without DSM-5 PTSD.**

**Figure 2:**
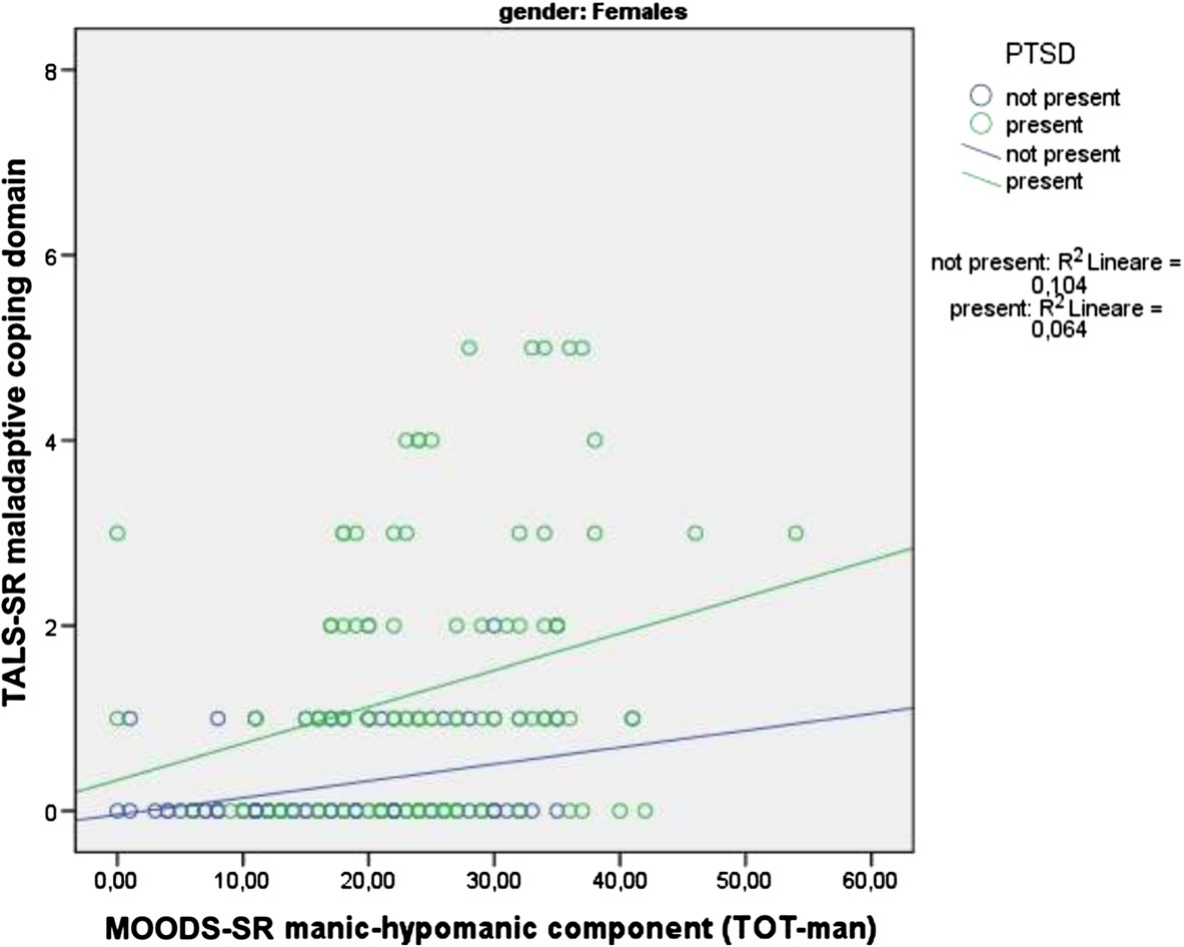
**Relationships between MOODS-SR manic-hypomanic component and TALS-SR maladaptive coping domain among females with and without DSM-5 PTSD.**

**Figure 3:**
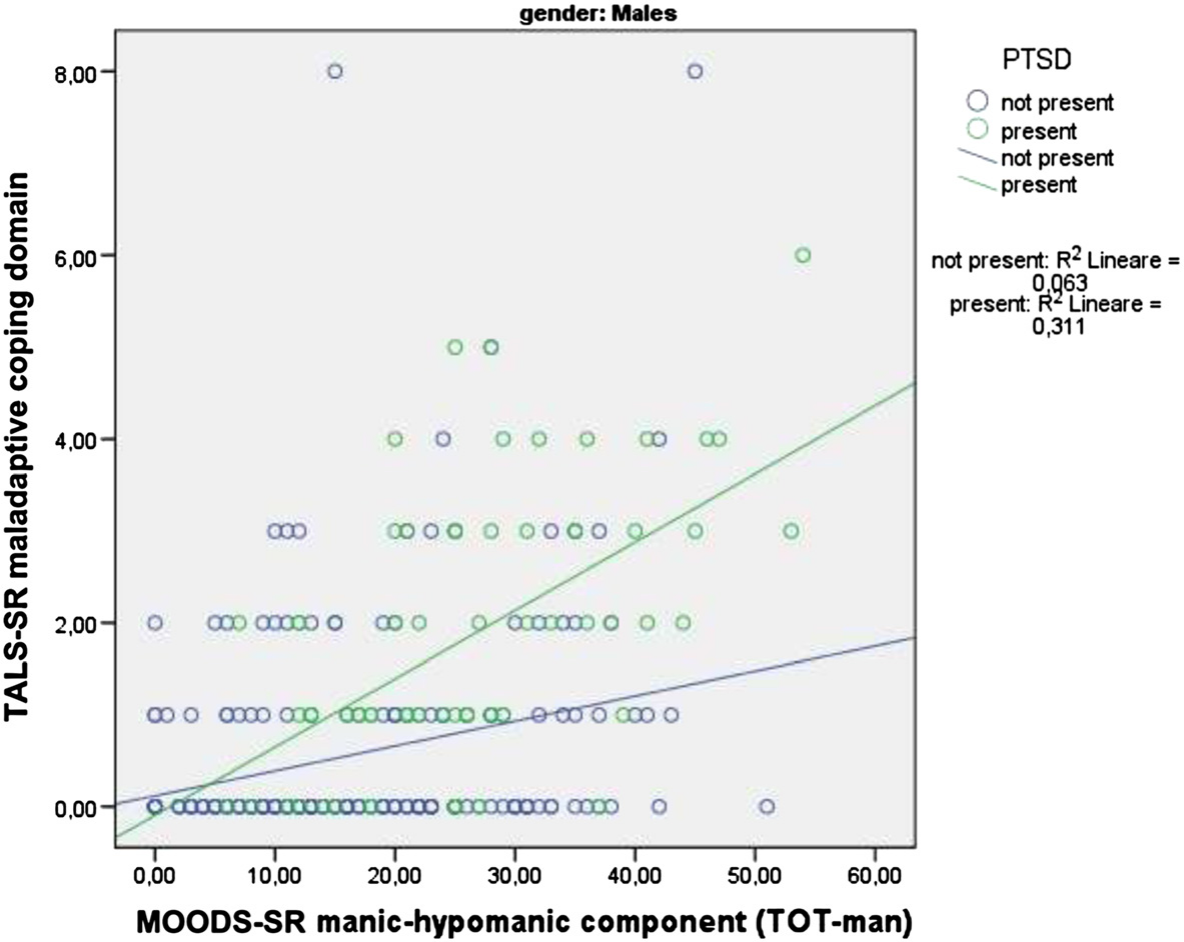
**Relationships between MOOD-SR manic-hypomanic component and TALS-SR maladaptive coping domain among males with and without PTSD.**

Two *t*-tests applied to the regression line slopes comparing survivors with PTSD with respect to those without, for what concern the association between the MOODS-SR manic-hypomanic component and the TALS-SR domains, showed a significantly stronger association with the *maladaptive coping* domain only [*b* =0.06 (SE =0.01) vs *b* =0.03 (SE =0.01), *t* =2.95, *p* < .010] (see scattergram in Figure 1) that was confirmed among males [*b* =0.07 (SE =0.01) vs *b* =0.03 (SE =0.01); *t* =2.99; *p* < .010] but not females [*b* =0.04 (SE =0.01) vs *b* =0.02 (SE =0.01); *t* =1.39; *p* = n.s.] (see scattergrams in Figures 2 and 3).

Computation of the point biserial correlation coefficients showed a significant “*moderate*” relationship between the MOODS-SR manic-hypomanic component and the TALS-SR maladaptive domain items *N* =97 (“*Stop taking care of yourself, for example not getting enough rest or not eating right?*”), *N* =98 (“*Stop taking prescribed medications or fail to follow up with medical recommendations…?*”), and *N* =100 (“*Engage in risk-taking behaviors…?*”) among survivors with PTSD.

Further, PTSD males showed a significant “*moderate*” correlation on TALS-SR items *N* =97 (“*Stop taking care of yourself, for example not getting enough rest or not eating right?*”), *N* =100 (“*Engage in risk-taking behaviors…?*”), and *N* =103 (“*Intentionally scratch, cut, burn, or hurt yourself…?*”) and also a significant “*good*” correlation on *N* =98 (“*Stop taking prescribed medications or fail to follow up with medical recommendations…?*”). Conversely, PTSD females showed a significant “*good*” correlation on item *N* =100 only (“*Engage in risk-taking behaviors…?*”).

A significantly higher correlation coefficient emerged in PTSD males than females for item *N* =98 only (“*Stop taking prescribed medications or fail to follow up with medical recommendations…?*”) (*r* = .410 vs *r* = .098, *z* =2.09, *p* = .040) (Table 2).

**Table 2:** **Point biserial correlation coefficients: association of TALS-SR maladaptive coping domain items and MOODS-SR manic-hypomanic component in the total sample of DSM-5 PTSD survivors and divided by gender**

**TALS-SR maladaptive items**		**PTSD total (** ***r*** **)**	**PTSD males (** ***r*** **)**	**PTSD females (** ***r*** **)**	***z***	***p***
97	*…Stop taking care of yourself, for example not getting enough rest or not eating right?*	.218*	.365*	.133	1.54	.12
98	*…Stop taking prescribed medications or fail to follow up with medical recommendations, such as appointments, diagnostic test or a diet?*	.256*	.410*	.098	2.09	.04
99	*… Use alcohol or drugs or over-the-counter medications to calm yourself or to relieve emotional or physical pain?*	.194*	.209	.172	0.24	.81
100	*…Engage in risk-taking behaviors, such as driving fast, promiscuous sex, hanging out in dangerous neighborhoods?*	.387*	.354*	.428*	−0.54	.59
101	*…Wish you hadn’t survived?*	.015	.061	−.031	0.57	.57
102	*…Think about ending your life?*	.188*	.192	.173	0.12	.90
103	*…Intentionally scratch, cut, burn, or hurt yourself?*	.148	.281*	−.004	1.81	.07
104	*…Attempt suicide?*	.102	.101	.091	0.06	.95

*p < .05.

The rates of endorsement of each TALS-SR maladaptive items in males and females with PTSD are reported in Table 3. Male survivors showed a significantly higher endorsement of TALS-SR maladaptive item 100 (“*Engage in risk-taking behaviors…?*”) than females.

**Table 3:** **Gender differences in the TALS-SR maladaptive coping items endorsement among DSM-5 PTSD survivors**

**TALS-SR maladaptive items**		**Total PTSD**	**PTSD males**	**PTSD females**	***χ*** ^**2**^	***p***
97	*…Stop taking care of yourself, for example not getting enough rest or not eating right?*	78(46.2%)	24(36.9%)	54(51.9%)	3.043	.081
*98*	*…Stop taking prescribed medications or fail to follow up with medical recommendations, such as appointments, diagnostic test or a diet?*	25(14.8%)	13(20.0%)	12(11.5%)	1.650	.199
*99*	*… Use alcohol or drugs or over-the-counter medications to calm yourself or to relieve emotional or physical pain?*	47(27.8%)	22(33.8%)	25(24.0%)	1.459	.227
100	*…Engage in risk-taking behaviors, such as driving fast, promiscuous sex, hanging out in dangerous neighborhoods?*	38(22.5%)	25(38.5%)	13(12.5%)	14.015	<.001
101	*…Wish you hadn’t survived?*	29(17.2%)	13(20.0%)	16(15.4%)	0.319	.572
102	*…Think about ending your life?*	16(9.5%)	9(13.8%)	7(6.7%)	1.606	.205
103	*…Intentionally scratch, cut, burn, or hurt yourself?*	16(9.5%)	9(13.8%)	7(6.7%)	1.606	.205
104	*…Attempt suicide?*	8(4.7%)	5(7.7%)	3(2.9%)	1.123	.289

## Discussion

To the best of our knowledge, this is the first study to explore gender differences in DSM-5 new maladaptive symptoms in a sample of earthquake survivors with PTSD diagnosed in accordance to DSM-5 criteria, besides their correlations and those of post-traumatic spectrum symptoms with lifetime mood spectrum symptoms.

Our results show significantly higher rates of endorsement in both the MOODS-SR manic and depressive domains in survivors with DSM-5 PTSD with respect to those without. Further, significant gender differences emerged among survivors with DSM-5 PTSD, both in the MOODS-SR depressive and rhythmicity and vegetative function components and in the TALS-SR loss events, reaction to losses, and re-experiencing and arousal domains. In all these cases, women reported significantly higher scores than men. Conversely, significantly higher rates emerged in men with respect to women for what concern the TALS-SR maladaptive domain only. These data are in line with previous literature highlighting gender differences in PTSD symptomatology diagnosed in accordance to DSM-IV-TR criteria with women usually reporting higher post-traumatic stress symptoms and men showing significantly higher rates of self-destructive or maladaptive behaviors [8,26,27,29-32,57].

Exploring the correlations between the MOODS-SR and the TALS-SR, a significant correlation emerged between the MOODS-SR depressive component and all TALS-SR domains among survivors with PTSD. Among the same subjects, a statistically significant correlation emerged between the MOODS-SR manic-hypomanic component and the TALS-SR potentially traumatic events and maladaptive coping domains only. These data corroborate previous research highlighting a correlation between bipolar disorder comorbidity and an increased risk for potentially traumatic exposure [34-36]. Otto et al. (2004), in fact, reported a 16% prevalence of PTSD in a sample of 1,214 patients with BD [35]. Among the risk factors for PTSD, bipolar subjects showed also the presence of greater trauma exposure. More recently, Pollack et al. (2006) studied 137 ongoing participants in the naturalistic, longitudinal study Systematic Treatment Enhancement Program for Bipolar Disorder (STEP-BD), who were indirectly exposed to September 11, 2001 terrorist attacks [36]. The authors reported in this sample a 20% PTSD prevalence rate. A new onset of PTSD was significantly associated with the presence of a hypomanic, manic, or mixed mood state at the time of trauma and mania/hypomania remained a significant predictor of PTSD in response to the September 11 attacks after controlling for peri-traumatic exposure and distress variables. Previous data on BD in PTSD highlighted not only high rates of such comorbidity but potential deleterious effects of trauma and PTSD on the course and outcome of BD and, conversely, of BD on the course and outcome of PTSD [39,58,59], recommending the need for further research. Our data corroborate these findings suggesting also a correlation between even subthreshold manic symptoms and an increased risk for maladaptive behaviors in PTSD.

The new symptom (E2) included in the DSM-5 PTSD criterion E (marked alterations in arousal and reactivity associated with the traumatic event), exploring reckless, maladaptive behaviors, addresses the important post-traumatic symptoms often seen in adolescents [29-32,60-63]. Friedman et al. (2011) suggested how the new DSM-5 criterion E encompasses more than the hyper-arousal addressed in previous DSM-IV-TR criterion D, better characterizing alterations in arousal and reactivity that are associated with the traumatic event [60]. Such a reframing of this symptom cluster enables us to include behavioral, as well as emotional indicators of such post-traumatic alterations. Our data are in line with the growing evidence that PTSD is associated with reckless and self-destructive behavior, particularly among young men. Israeli male adolescents, exposed to recurrent terrorism, exhibited marked increases in risk-taking behavior [15]. Reckless driving was observed among both adult individuals and adolescents with PTSD [26,64-67]. Risky sexual behaviors, sometimes associated with HIV risk was reported among college women, female prisoners, and adult male survivors of childhood sexual abuse [65,68]. Reckless behavior appears to be associated with PTSD to such an extent that it has been added to the diagnostic cluster assessing alterations in arousal and reactivity. It is of interest that our data seem to suggest a relationship between these symptoms and a bipolar diathesis particularly among young men with PTSD that should need further attention. Exploring the gender differences in the endorsement of the TALS-SR maladaptive domain items among survivors with PTSD, our results showed a statistically significant gender difference for what concern the item 100 only (“*Engage in risk-taking behaviors…?*”), with males reporting the highest rates. Looking at the point biserial correlation coefficients exploring the relationships between the MOODS-SR manic-hypomanic component and the TALS-SR maladaptive domain items, stronger correlations were confirmed in males. Our results seem to confirm the gender relevance outlined in the introduction section of DSM-5. The new manual points out the possible influence of gender not only on the overall risk for the development of a mental disorder, as shown by prevalence and incidence rates, but also on the likelihood of particular symptoms and consequently on the need of service provision.

Interpretation of our results should keep in mind some important limitations of the study. First, the most important is the use of self-report instruments that may be considered less accurate. Nevertheless, the use of TALS-SR allowed us to accurately compare the possible DSM-IV-TR and DSM-5 criteria reported by the earthquake survivors. Second, as already mentioned in a previous study [10], is the lack of information on the presence of Axis I psychiatric comorbidities and the lack of assessment on the functional impairment. Third is the homogeneity of the study sample that included non-clinical high school students. Fourth, the MOODS-SR lifetime version did not permit to establish if the manic-hypomanic component was present before the onset of PTSD or emerged after it, so that no direction of causality could be determined. Further studies in larger samples of earthquake-exposed subjects are thus needed in order to confirm our results, particularly for what concern the gender differences.

## Conclusion

Despite the abovementioned limitations, this study offers an important glimpse at the empirical performance of the DSM-5 PTSD criteria, suggesting the need for further studies in epidemiological samples to evaluate the change in prevalence rates of PTSD that may result by adopting the new DSM-5 criteria.

## Abbreviations

PTSD: Post-traumatic stress disorder
DSM: Diagnostic and Statistical Manual
BD: Bipolar disorder
TALS-SR: Trauma and Loss Spectrum-Self Report
MOODS-SR: Mood Spectrum-Self Report
STEP-BD: Systematic Treatment Enhancement Program for Bipolar Disorder
NCS: National Comorbidity Survey
